# Urological symptoms among 23,240 men in the general danish population – concerns about symptoms, their persistence and influence on primary care contacts

**DOI:** 10.1080/02813432.2018.1487377

**Published:** 2018-07-25

**Authors:** Majken Solvang, Sandra Elnegaard, Dorte Ejg Jarbøl

**Affiliations:** a University of Southern Denmark;; b Research Unit of General Practice, University of Southern Denmar

**Keywords:** Help-seeking behaviour, patient acceptance of health care, urination, urinary incontinence, symptom assessment, general practice

## Abstract

**Objective:** To analyse possible associations between men’s likelihood of contacting a general practitioner (GP) for urological symptoms and the persistence of the symptoms, the influence on daily activities and the level of concern about the symptoms.

**Design:** Web-based nationwide cross-sectional questionnaire study.

**Setting:** The general population in Denmark.

**Subjects:** 48,910 randomly selected men aged 20+ years.

**Main outcome measures:** Urological symptom prevalence and odds ratios for GP contact with urological symptoms in regard to concern for the symptom, influence on daily activities and the persistence of the symptom.

**Results:** Some 23,240 men responded to the questionnaire, yielding a response rate of 49.8%. The prevalence of at least one urological symptom was 59.9%. Among men experiencing at least one urological symptom almost one-fourth reported contact to general practice regarding the symptom. Approximately half of the symptoms reported to be extremely concerning were discussed with a GP.

**Conclusion:** Increased symptom concern, influence on daily activities and long-term persistence increased the likelihood of contacting a GP with urological symptoms. This research points out that guidelines for PSA testing might be challenged by the high prevalence of urological symptoms.

**Key points**
 The decision process of whether to contact the general practitioner (GP) is influenced by different factors, but contradictory results has been found in triggers and barriers for help-seeking with urological symptoms.
  • Increased symptom concern, influence on daily activities and long-term persistence consistently increased the likelihood of contacting a general practitioner with urological symptoms in men.
  • Only 50% of the symptoms reported to be extremely concerning were however discussed with the GP.
  • Guidelines for PSA testing might be challenged by the high prevalence of urological symptoms.

## Introduction

The decision process of whether to contact the general practitioner (GP) is influenced by different factors, and symptoms presented to the GP represent only an extract of the total symptom pool experienced by individuals in the general population [1]. Knowledge of triggers and barriers for healthcare seeking is therefore important in order to enhance the ability to early diagnosis and prompt treatment.

Urological symptoms are frequent in the general population and often perceived as a normal part of ageing which might prevent individuals from consulting a GP [2–4]. The prevalence of urological symptoms varies however considerably in different studies. Urinary incontinence (UI) among men has been reported to be between 4.3-16.2% [5–7] and the prevalence of lower urinary tract symptoms (LUTS) among men to be 39.1-83% [7–11]. The wide range is presumably due to different definitions, methods and study populations. The prevalence for medical help-seeking for urological symptoms also varies in the literature with ranges of GP contact regarding urological symptoms among men 5.6-17.5% [5,6,12].

Generally, urological symptoms are of benign origin and cover the base of the symptom pyramid, where the top could be a sign of advanced urological cancers [13]. However, symptoms of benign prostatic hyperplasia (BPH) are largely the same as those of prostate cancer and the symptoms can relatively easily be further examined by a GP, for instance by using digital rectal exam and prostate specific antigen (PSA) test. Since prostate cancer is the most frequently diagnosed cancer among men in Denmark, second most diagnosed worldwide, it is important to further investigate the factors that determine whether or not an individual contacts a GP when experiencing urological symptoms [14].

Reasons for non-attendance with urological symptoms might be explained by the perception of the symptoms. Urological symptoms are subject to stigmatization; especially UI and the odour of urine cause this stigmatization [15]. Along the same line, embarrassment and shame are feelings found to be associated with frequent urination and UI stad [3,15].

The perception of symptoms can be studied with numerous different variables such as bother, concern, worry, tension, nervousness and anxiety. Using a reflecting model all these variables can be used to measure psychological distress [16]. Several studies found an association between self-rated bother due to urological symptoms and help-seeking [6,12,17]. However, when looking at different degrees of bother Sladden et al. found that increased self rated symptom-bother did not lead to increased GP-attendance [18]. Besides bother, anxiety about cancer has been identified as predictors for help-seeking [18,19]. Prior studies have used relatively small sampling frames, and it is therefore interesting to investigate how concern and influence on daily activity affects the help-seeking behaviour on a larger and population-based scale.

The aim of this study was therefore to analyse possible associations between men’s likelihood of contacting a general practitioner for urological symptoms and the persistence of the symptoms, the influence on daily activities and the level of concern about the symptoms.

## Material and methods

### Study design and population

**Figure 1.**
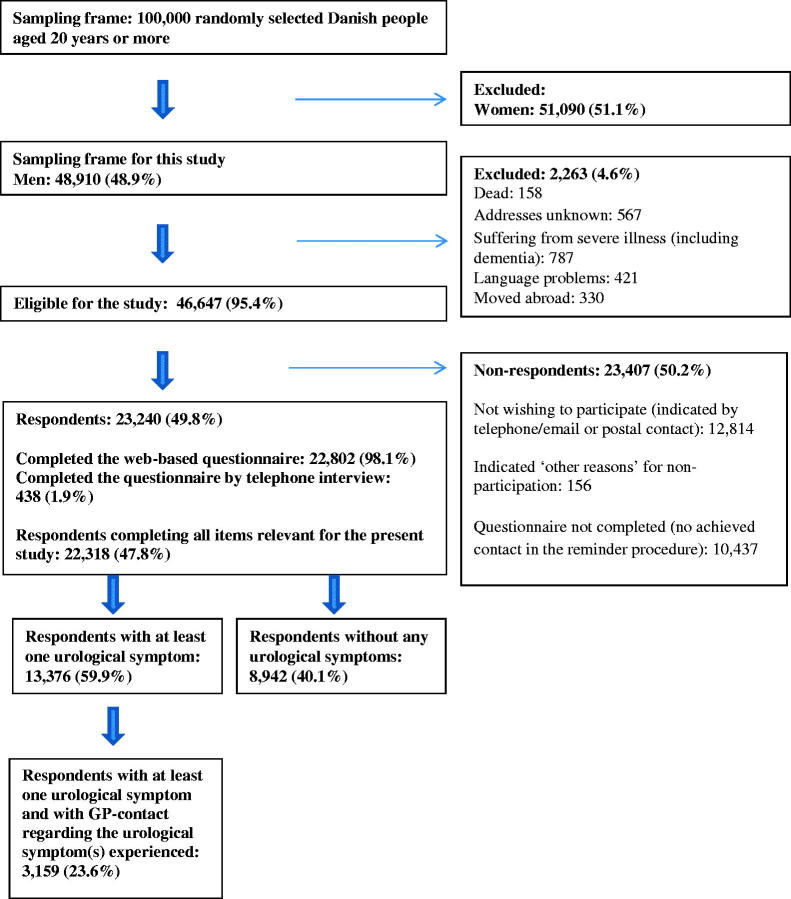
Study cohort.

As part of The Danish Symptom Cohort (DaSC) [20], this nationwide cross-sectional study was conducted. A comprehensive web-based questionnaire was distributed to 100,000 people, aged 20+ years, randomly selected via the Danish Civil Registration System, which contains a unique personal identification number for all Danish citizens [21]. In this study, we included only the men (Figure 1). A postal letter explaining the purpose of the study and a unique 12-digit login was sent to all individuals in the study sample. The non-respondents received a reminding letter after two weeks. Another two weeks later, the non-respondents were contacted by telephone and encouraged to participate. Those without a computer, smartphone or tablet were offered the opportunity to answer the questionnaire as a telephone interview [20]. The data collection took place between June and December 2012.

### Questionnaire

The questionnaire contained 44 different symptoms, covering cancer alarm symptoms and some frequently occurring symptoms throughout the body [20]. The wording of the question regarding symptom experience was:” have you experienced any of the following sensations, symptoms or discomfort within the past 4 weeks?” [1]. In this study, we only included items regarding the ten urological symptoms. All ten urological symptoms appear from Table 2. Most of the urological symptoms studied are included in the definition LUTS. They consist of storage symptoms (night-time urination, frequent urination, incontinence with or without stress/urge), and voiding symptom (difficulty in emptying the bladder). Moreover, we included blood in urine, blood in semen and erectile dysfunction.

For each individual symptom the participants had experienced, they were, among other things, asked about their concern for the symptom, when they first experienced the symptom, the degree of influence on their daily activities, and whether or not they contacted their GP in person, by phone or by e-mail, with regard to the symptom. Furthermore, they were asked about general concern for their current health. For items regarding blood in semen and erective dysfunction the respondents had the opportunity to answer: “Do not wish to answer”. In that case symptoms were excluded from the analysis and accounted as missing.

### Statistical analysis

We constructed two different data sets from the questionnaire responses, one compiling all urological symptom experiences into one group, another looking at factors influencing each individual when experiencing urological symptoms.

We calculated how symptoms were distributed and the frequency of GP contact in the following age groups: 20–39, 40–59, 60–79 and ≥80 years.

The following covariates were considered in the analysis: symptom concern, general concern, level of influence on daily activities, symptom persistence and symptom burden. Concern and level of influence on daily activities were categorised on a five-point Likert scale: “not at all”, “slightly”, “moderate”, “quite a bit” and “extremely”. Symptom persistence was categorised based on when the respondents first experienced the symptom, and was divided into 4 groups: <1 month, 1–3 months, 3-6 months, >6 months. Persistence >6 months is referred to as long-term. Finally, a variable used as a proxy for the urological symptom burden was constructed as the number of urological symptoms experienced by each individual. The urological symptom burden was categorised into five groups 0, 1, 2–3, 4–5, ≥6. Calculations estimating the number of symptom experiences and the proportion of symptoms with GP contact for each of the covariates except urological symptom burden were made.

To evaluate collinearity between symptom concern, symptom persistence, influence on daily activity, and general concern, correlation coefficients were calculated with Spearman’s rank correlation.

Number of urological symptoms and the proportions of symptoms leading to GP-contact were calculated. Logistic regression models were used to calculate the crude and adjusted odds ratios (ORs) for associations between concern, symptom persistence, symptom burden and influence on daily activities and GP contact with any urological symptoms and stratified on symptom type, respectively. Adjustments were made for possible confounders: age and covariates.

Confidence intervals were calculated using the binomial distribution, p-value below 0.05 was considered statistically significant, and data analyses were carried out using StataIC 13**^©^**.

## Results

Among the 100,000 individuals receiving the questionnaire, 48,910 were male. Some 23,240 men completed the questionnaire, yielding a response rate among the men of 49.8%. A total of 22,318 men completed all relevant items for the present study and were included for analyses. The overall prevalence of at least one self-reported urological symptoms in the four weeks period was 59.9%. **S**ome 23.6% reported contact to a GP with at least one of the symptom(s) experienced. (Figure 1).

In total 23,070 urological symptoms were reported. The highest proportion of urological symptoms were reported among individuals in the oldest age groups, and some 68.8% of the urological symptoms were experienced for the first time more than six months ago, Table 1. Contacting a GP with urological symptoms was much less common in the two youngest age groups (9.9% and 17.1%, respectively). The proportion of urological symptoms leading to GP contact was highest among symptoms reported as extremely concerning (50.4%) and with an extreme degree of influence on daily activities (46.9%), Table 1.

**Table 1.** The top half shows some of the characteristics of the study sample. The bottom half provides an overview of self-reported urological symptom experiences with regard to the three covariates and GP contact.

	Study sample		Number of urological symptoms		Number of urological symptoms with GP-contact	
	n	(%)	n	(%)	n	(%)
Study sample						
Overall	22 318	(100.0)	23 070	(100.0)	5 632	(24.4)
Age						
20–39	5 311	(23.8)	2 345	(10.2)	232	(9.9)
40–59	8 875	(39.8)	7 570	(32.8)	1 293	(17.1)
60–79	7 531	(33.7)	11 924	(51.7)	3 695	(31.0)
80+	601	(2.7)	1 231	(5.3)	412	(33.5)
Number of urological symptoms. Referred to as urological symptom burden						
0	8 942	(40.1)	–	–	–	–
1	7 334	(32.9)	7334	(31.8)	844	(11.5)
2–3	5 161	(23.1)	11809	(51.2)	3103	(26.3)
4–5	788	(3.5)	3340	(14.5)	1371	(41.0)
≥6	93	(0.4)	587	(2.5)	314	(53.5)
Time since first experience of the symptom. Referred to as symptom persistence						
<1 month			4 436	(19.2)	833	(18.8)
1–3 months			1 245	(5.4)	237	(19.0)
3–6 months			1 511	(6.5)	306	(20.3)
>6 months			15 878	(68.8)	4 256	(26.8)
Influence on daily activities						
Not at all			5 769	(25.0)	691	(12.0)
Slightly			7 079	(30.7)	1 286	(18.2)
Moderate			4 881	(21.2)	1 436	(29.4)
Quite a bit			3 353	(14.5)	1 286	(38.4)
Extremely			1 988	(8.6)	933	(46.9)
Concern for the symptom						
Not at all			8 999	(39.0)	1 101	(12.2)
Slightly			6 540	(28.3)	1 550	(23.7)
Moderate			3 705	(16.1)	1 210	(32.7)
Quite a bit			2 360	(10.2)	1 032	(43.7)
Extremely			1 466	(6.4)	739	(50.4)

The most frequently occurring urological symptom was night-time urination with a prevalence of 47.9%, while the most infrequent symptoms were blood in urine (0.5%) and blood in semen (0.4%). The proportion of urological symptoms leading to GP contact ranged from 16.9-69.0%, for night-time urination and blood in urine, respectively (Table 2). GP contact with urological symptoms was consistently higher among men aged 60 years and older, Table 2.

**Table 2.** Number of urological symptoms and number of symptoms with GP-contact stratified on age (above/below 60 years).

	Number of symptoms N = 23 070			Number of symptoms with GP-contact			Number of symptoms <60 years N = 9 915			Number of symptoms with GP-contact <60 years			Number of symptoms ≥=60 years N = 13 155			Number of symptoms with GP-contact ≥ =60 years		
Symptom	n	(%)	CI 95%	n	(%)	CI 95%	n	(%)	CI 95%	n	(%)	CI 95%	n	(%)	CI 95%	n	(%)	CI 95%
Night-time urination	11055	(47.9)	47.3-48.6	1866	(16.9)	16.2-17.6	5339	(53.8)	52.9-54.8	470	(8.8)	8.0-9.6	5716	(43.5)	42.6-44.3	1396	(24.4)	23.3-25.5
Erectile dysfunction	4103	(17.8)	17.3-18.3	1317	(32.1)	30.7-33.5	1134	(11.4)	10.8-12.1	352	(31.0)	28.3-33.7	2969	(22.6)	21.9-23.3	965	(32.5)	30.8-34.2
Difficulty in emptying the bladder	3222	(14.0)	13.5-14.4	969	(30.1)	28.5-31.7	1363	(13.7)	13.1-14.4	261	(19.1)	17.1-21.2	1859	(14.1)	13.5-14.7	708	(38.1)	35.9-40.3
Frequent urination	2465	(10.7)	10.3-11.1	706	(28.6)	26.9-30.4	1243	(12.5)	11.9-13.2	208	(16.7)	14.7-18.8	1222	(9.3)	8.8-9.8	498	(40.8)	38.0-43.5
Stress incontinence	235	(1.0)	0.9-1.1	85	(36.2)	30.0-42.4)	78	(0.8)	0.6-1.0	21	(26.9)	16.9-37.0	157	(1.2)	1.0-1.4	64	(40.8)	33.0-48.5
Urge incontinence	1117	(4.8)	4.6-5.1	310	(27.8)	25.1-30.4	364	(3.7)	3.3-4.0	69	(19.0)	14.9-23.0	753	(5.7)	5.3-6.1	241	(32.0)	28.7-35.3
Incontinence without stress/urge	305	(1.3)	1.2-1.5	108	(35.4)	30.0-40.8	93	(0.9)	0.7-1.1	27	(29.0)	19.6-38.4	212	(1.6)	1.4-1.8	81	(38.2)	31.6-44.8
Pain/burning when urinating	363	(1.6)	1.4-1.7	148	(40.8)	35.7-45.9	210	(2.1)	1.8-2.4	72	(34.3)	27.8-40.8	153	(1.2)	1.0-1.3	76	(49.7)	41.7-57.7
Blood in urine	116	(0.5)	0.4-0.6	80	(69.0)	60.4-77.5	52	(0.5)	0.4-0.7	32	(61.5)	47.9-75.2	64	(0.5)	0.4-0.6	48	(75.0)	64.1-85.9
Blood in semen	89	(0.4)	0.3-0.5	43	(48.3)	37.7-58.9	39	(0.4)	0.3-0.5	13	(33.3)	17.9-48.8	50	(0.4)	0.3-0.5	30	(60.0)	45.9-74.1

Increased symptom concern, influence on daily activity and long-term persistence were all associated with GP-attendance with urological symptoms. A further determinant for GP contact was the urological symptom burden, (OR 5.86, CI 4.06-8.46) in the group with experience of six or more urological symptoms. Contrarily, short-term symptom persistence (three to six months) was associated with lower odds for GP contact, OR 0.76, 95 CI 0.62-0.92, Table 3.

**Table 3.** Odds Ratios (ORs) for GP contact for all reported urological symptoms (*n* = 23 070) with regard to symptom concern, influence on daily activities, symptom persistence and symptom burden.

	n	*OR crude*	CI (95%)	*OR adj.*	CI (95%)
Time since first experience of the symptom. Referred to as symptom persistence*					
<1 month	4 436	1.00	ref	1.00	ref
1–3 months	1 245	1.02	(0.83-1.25)	0.87	(0.70-1.07)
3–6 months	1 511	1.10	(0.91-1.33)	**0.76**	(0.62-0.92)
>6 months	15 878	**1.58**	(1.41-1.78)	**1.23**	(1.09-1.40)
Influence on daily activities**					
Not at all (ref.)	5 769	**1.00**	(1.00-1.00)	**1.00**	(1.00-1.00)
Slightly	7 079	**1.63**	(1.45-1.83)	**1.42**	(1.26-1.60)
Moderate	4 881	**3.06**	(2.72-3.45)	**2.33**	(2.05-2.64)
Quite a bit	3 353	**4.57**	(4.02-5.20)	**3.13**	(2.73-3.59)
Extremely	1 988	**6.50**	(5.59-7.56)	**3.99**	(3.40-4.68)
Concern for the symptom**					
Not at all (ref.)	8 999	1.00	–	**1.00**	–
Slightly	6 540	**2.22**	(2.00-2.47)	**1.83**	(1.64-2.05)
Moderate	3 705	**3.53**	(3.14-3.97)	**2.68**	(2.36-3.05)
Quite a bit	2 360	**5.58**	(4.90-6.34)	**3.98**	(3.46-4.58)
Extremely	1 466	**7.33**	(6.27-8.58)	**4.80**	(4.05-5.68)
Number of urological symptoms. Referred to as urological symptom burden*					
1	7334	1.00	(1.00-1.00)	1.00	(1.00-1.00)
2–3	11809	**2.74**	(2.50-3.00)	**2.25**	(2.04-2.47)
4–5	3340	**5.35**	(4.65-6.17)	**3.90**	(3.35-4.54)
≥6	587	**8.84**	(6.25-12.52)	**5.86**	(4.06-8.46)

Bold indicates P-value <0.05*Adjusted for general concern, concern for the symptom, influence on daily activities, age, urological burden and persistence.

** Adjusted for general concern, urological burden, persistence and age.

The odds ratios for GP contact with regard to each urological symptom are shown in Table 4. A similar pattern as described above was found: The likelihood for GP contact increased with increasing influence on daily activities, increasing concern and long-term persistence, and varied inconsiderably among the symptoms. For pain/burning when urinating, the likelihood for GP contact was however decreased (OR 0.36, 95 CI 0.14-0.91) when the symptom was experienced for the first time between three and six months ago, Table 4.

**Table 4.** Odds Ratios (ORs) for GP contact for reported urological symptoms with regard to symptom concern, influence on daily activities and symptom persistence, stratified on symptom type. N = number of symptoms.

	Night-time urination N = 11424			Erectile dysfunction N = 4289			Pain/burning when urinating N = 384			Frequent urination N = 2597			Stress incontinenceN =256		
	*OR*	*OR adj.*	CI (95%)	*OR*	*OR adj.*	CI (95%)	*OR*	*OR adj.*	CI (95%)	*OR*	*OR adj.*	CI (95%)	*OR*	*OR adj.*	CI (95%)
Time since first experience of the symptom. Referred to as symptom persistence*															
<1 month	1.00	ref	–	1.00	Ref	–	1.00	ref	–	1.00	ref	–	1.00	ref	–
1–3 months	0.86	0.72	(0.49-1.06)	**0.64**	**0.61**	(0.40-0.93)	1.12	1.02	(0.49-2.12)	0.83	0.75	(0.52-1.08)	0.17	0.14	(0.01-1.40)
3–6 months	1.16	0.79	(0.58-1.09)	0.74	**0.70**	(0.50-0.99)	0.55	**0.36**	(0.14-0.91)	1.22	0.80	(0.55-1.15)	0.61	0.71	(0.21-2.45)
>6 months	**1.76**	**1.34**	(1.15-1.57)	**1.26**	**1.24**	(1.01-1.52)	1.05	0.82	(0.46-1.44)	**2.34**	**1.35**	(1.05-1.74)	1.14	0.90	(0.37-2.17)
Influence on daily activities**															
Not at all (ref.)	1.00	ref	–	1.00	Ref	–	1.00	ref	–	1.00	ref	–	1.00	ref	–
A little bit	**1.83**	**1.53**	(1.31-1.78)	1.05	1.10	(0.85-1.43)	1.41	1.07	(0.54-2.10)	**1.49**	1.43	(0.96-2.13)	1.93	1.81	(0.56-5.89)
Somewhat	**3.72**	**2.63**	(2.23-3.10)	**1.64**	**1.66**	(1.31-2.11)	**2.79**	**2.24**	(1.06-4.74)	**3.24**	**2.61**	(1.76-3.87)	2.76	**3.68**	(1.02-13.24)
Quite a bit	**5.17**	**3.15**	(2.62-3.80)	**2.30**	**2.33**	(1.84-2.94)	**4.97**	**3.88**	(1.60-9.41)	**5.76**	**4.09**	(2.70-6.18)	4.58*	**3.75**	(1.07-13.17)
Extremely	**7.15**	**3.88**	(3.03-4.97)	**2.91**	**2.90**	(2.29-3.66)	**6.19**	**4.23**	(1.49-11.96)	**10.20**	**7.20**	(4.38-11.82)	**18.72**	**14.27**	(3.02-67.49)
Concern for the symptom**															
Not at all (ref.)	1.00	ref	–	1.00	Ref	–	1.00	ref	–	1.00	ref	–	1.00	ref	–
A little bit	**2.61**	**1.95**	(1.71-2.24)	1.17	1.22	(0.97-1.53)	2.23	1.96	(0.86-4.50)	**2.11**	**1.73**	(1.28-2.32)	1.60	1.26	(0.53-2.98)
Somewhat	**4.56**	**3.09**	(2.61-3.65)	**1.60**	**1.67**	(1.33-2.10)	**4.37**	**4.18**	(1.70-10.29)	**3.73**	**2.79**	(2.02-3.83)	2.07	1.86	(0.67-5.15)
Quite a bit	**6.63**	**3.93**	(3.20-4.83)	**2.28**	**2.35**	(1.88-2.93)	**8.75**	**7.95**	(3.01-21.03)	**6.37**	**4.15**	(2.87-6.00)	**4.37**	**4.63**	(1.34-15.98)
Extremely	**9.35**	**5.13**	(3.82-6.88)	**2.79**	**2.82**	(2.24-3.55)	**12.49**	**11.33**	(3.61-35.60)	**12.44**	**7.88**	(4.85-12.79)	**21.25**	**18.28**	(3.63-92.10)
	Urge incontinence N = 1184			Incontinence without stress/urge N = 328			Difficulty in emptying the bladder N = 3365			Blood in urine N = 125			Blood in semen N = 94		
	*OR*	*OR adj.*	CI (95%)	*OR*	*OR adj.*	CI (95%)	*OR*	*OR adj.*	CI (95%)	*OR*	*OR adj.*	CI (95%)	*OR*	*OR adj.*	CI (95%)
Time since first experience of the symptom. Referred to as symptom persistence*															
<1 month	1.00	ref	–	1.00	Ref	–	1.00	ref	–	1.00	ref	–	1.00	ref	–
1–3 months	1.53	1.29	(0.66-2.52)	0.94	1.06	(0.23-4.79)	0.72	0.70	(0.45-1.07)	2.74	2.59	(0.72-9.32)	2.00	1.44	(0.33-6.31)
3–6 months	1.36	1.13	(0.62-2.06)	0.78	0.61	(0.19-1.99)	0.78	**0.68**	(0.47-0.98)	***			4.00	2.91	(0.62-13.61)
>6 months	**2.81**	**2.50**	(1.62-3.84)	**2.27**	**2.17**	(1.07-4.43)	**1.58**	1.22	(0.98-1.53)	1.76	1.37	(0.44-4.30)	2.43	2.06	(0.67-6.35)
Influence on daily activities**															
Not at all (ref.)	1.00	ref	–	1.00	Ref	–	1.00	ref	–	1.00	ref	–	1.00	ref	–
A little bit	1.21	1.13	(0.60-2.13)	1.50	1.54	(0.45-5.31)	1.17	1.09	(0.84-1.42)	2.18	2.02	(0.64-6.40)	1.32	1.97	(0.50-7.82)
Somewhat	**2.11**	1.74	(0.93-3.28)	3.19	3.12	(0.89-10.87)	**1.92**	**1.62**	(1.24-2.13)	1.94	1.99	(0.50-7.91)	**4.79**	**8.42**	(1.61-44.11)
Quite a bit	**3.33**	**2.59**	(1.36-4.94)	2.43	2.22	(0.63-7.85)	**3.16**	**2.57**	(1.91-3.46)	2.33	1.49	(0.26-8.46)	4.79	6.39	(0.74-55.32)
Extremely	**5.45**	**3.79**	(1.90-7.56)	**3.94**	2.97	(0.81-10.84)	**5.13**	**3.99**	(2.69-5.92)	2.59	2.50	(0.49-12.79)	**9.58**	**16.39**	(1.09-245.94)
Concern for the symptom**															
Not at all (ref.)	1.00	ref	–	1.00	Ref	–	1.00	ref	–	1.00	ref	–	1.00	ref	–
A little bit	1.31	1.18	(0.74-1.87)	1.05	0.93	(0.41-2.11)	**1.26**	1.24	(0.99-1.55)	1.75	0.88	(0.20-3.96)	2.26	2.26	(0.31-16.54)
Somewhat	**1.81**	1.48	(0.90-2.42)	0.65	0.53	(0.22-1.29)	**1.79**	**1.72**	(1.34-2.21)	1.90	1.09	(0.21-5.59)	**7.00**	**10.88**	(1.20-98.75)
Quite a bit	**2.97**	**2.09**	(1.23-3.55)	1.80	1.57	(0.65-3.80)	**3.87**	**3.51**	(2.60-4.74)	3.35	2.44	(0.50-11.88)	7.00	**16.09**	(1.36-190.58)
Extremely	**6.92**	**5.12**	(2.84-9.25)	2.29	1.63	(0.63-4.23)	**3.83**	**3.34**	(2.20-5.06)	2.10	2.49	(0.43-14.41)	**9.33**	**38.39**	(2.19-673.98)

Bold indicates P-value <0.05.

* Adjusted for general concern, concern for the symptom, influence on daily activities, age, urological burden and symptom persistence.

** Adjusted for general concern, urological symptom burden, symptom persistence and age.

*** Analysis not possible due to few observations.

## Discussion

### Summary of main findings

In this nationwide study among men in Denmark it is demonstrated that the prevalence of urological symptom is common, six of ten respondents reported at least one urological symptom within four weeks. Almost one-fourth reported contact to a GP regarding the symptom(s) experienced. Increased symptom concern, influence on daily activities and long-term persistence increase the likelihood of contacting a general practitioner with urological symptoms. However, only half of the symptoms reported to be extremely concerning were discussed with a GP.

### Strengths and limitations of the study

This study is population-based and includes 23,240 male respondents, which, to our knowledge, is the largest population-based study in this field up to now. The response rate was comparable to other population-based studies [8,22]. Respondents were slightly younger than non-respondents, but otherwise fairly representative of the Danish population [1]. However, under- or overestimation of prevalence cannot be refuted, since willingness to answer the questionnaire might be associated with the presence of symptoms. Both numerous symptoms and no symptoms at all could cause individuals to refrain from replying, which is why the role of non-respondents is uncertain. The participants were given the opportunity to answer the questionnaire by telephone, therefore, computer access and reading skills were unnecessary for participation.

Recall bias is inevitable since we are dealing with self-reported symptoms experienced in the preceding four weeks, and GP contact whenever. Some individuals will have reported symptoms experienced outside the four weeks timeframe, others will have forgotten to report experienced symptoms [23], but, given the limited timeframe for experienced symptoms, we believe that recall bias is kept to a minimum and plays a minor role.

In this study the category ‘Night-time urination’ covers anyone who checkmarked “That you have to get up to urinate at night”, while other comparable studies use the symptom category ‘nocturia’ defined as urinating at least twice during night-time. Furthermore, standardised scales like International Prostate Symptom Score and International Consultation on Incontinence Questionnaire were not included in the questionnaire used for the present study. Thus the prevalence of urological symptoms might not be completely comparable to other studies.

### Discussion of findings and existing literature

The prevalence of urological symptoms was indeed somewhat lower (59.9%) than previously reported. Coyne et al. [24] found the prevalence of LUTS to be 72%, but studied men aged 40 years or above. Additionally explanations for different numbers could therefore be that we did not include symptoms as terminal dripping and weak stream, and that the prevalence of urological symptoms increases with increasing age [25]. A recent study reported an even higher prevalence of urological symptoms (80%) among men aged 18 years or above [7]. With almost the same age group as in the present study, and nocturia defined as urinating at least once during night-time equivalent to night-time urination in the present study, the most likely explanation for a higher prevalence of urological symptoms is due to additional LUTS symptoms in the Kogan et al. study [7]. A large population-based study in the UK reported a 1-year period prevalence of UI to be 14.2% [26]. This prevalence is considerably higher than the one we found (7.7%). However, different time frames might explain this difference.

We found that when respondents reported the first experience of a urological symptom to be one to six months ago it decreased the likelihood of contacting a GP regarding the symptom. A possible explanation for the findings could be that people either seek help immediately after experiencing a symptom if they suspect an infection or a serious disease, or wait a while (more than six months according to our study) in order to see if the symptom alleviates on its own. Patients can have different expectations when consulting a GP. Sometimes patients are worried about serious illness and expect a referral to further examinations, at other times patients expect a prescribed medication [27]. These expectations from the patients are presumably dependent on the symptom(s). The present results for ‘pain/burning when urinating’ substantiates this point since pain or burning when urinating most often are a sign of a urinary tract infection [28] and for this particular symptom, the odds for GP contact were lower with symptom persistence of three to six months, probably because the men were not concerned about the symptom. These considerations are however hypothetical and should be examined in detail in future studies.

Contradicting results regarding triggers and barriers for healthcare seeking with urological symptoms are reported in the literature. Cunningham-Burley et al. [9] reported that suspected urinary infection, pain or persistent urinary symptoms were the most common reasons for GP contact. And that symptoms interfering with daily life did not cause concern and was not considered a reason for GP contact. These observations contradict the findings in the present study of an association between symptom concern, influence on daily activities and GP contact. McGrother et al. [26] found however a strong association between self-reported quality of life, which they defined by several factors including influence on daily activities, and increased help seeking with urological symptoms.

Depending on symptom type and location fear plays an important role in help-seeking, acting as an incentive for some people and as a hindrance for others [29]. For instance, Sladden et al showed that anxiousness about prostate cancer was associated with GP contact [18]. The definition of fear about cancer is not quite comparable to our self-reported symptom concern; however they do somewhat corroborate our results.

In our study almost two-thirds reported experience of urological symptoms within the past four weeks. The high prevalence of urological symptoms could be a challenge for the current guidelines for PSA testing because most of the symptoms are of benign origin or are due to cancer were there is no need for active treatment at the moment. From the literature we know that the positive predictive value (PPV) of LUTS to predict prostate cancer is very low due to the high prevalence of LUTS in the general population compared to a relatively low prostate cancer incidence [14]. NICE guideline states that any experienced LUTS or erectile dysfunction or haematuria should cause the GP to consider doing a PSA test. ‘Consider’ is used in the guidelines when confident that the intervention “will do more good than harm for most patients” [30]. Similarly the Danish cancer referral guideline for prostate cancer states that men experiencing LUTS symptoms or several incidents of haematospermia should be offered a PSA test, however, the PSA test should not be used for screening purposes [31]. Age is not a specific criterion in the guidelines. However, the risk of cancer is increasing with increasing age and should also form part of the decision. In the present study, the number of urological symptoms with GP contact was consistently higher among men aged 60 years or above. For difficulty in emptying the bladder, night-time urination and frequent urination the GP-contact was more than twice as high compared to men below 60 years of age. The literature points to the case that an increase in consultation frequency actually is observed 80–100 days prior to a prostate cancer diagnosis [32]. This could suggest that to some degree, individuals experiencing urological symptoms distinguish well between whether or not to contact a GP .However it is out of the boundaries of this study to answer that question.

## Conclusion and implications

We found that increasing symptom concern, influence on daily activities and long-term persistence was associated with a higher likelihood of contacting a general practitioner with regard to a urological symptom in men. However, only half of the symptoms reported to be extremely concerning were discussed with a GP, possibly due to stigmatization, embarrassment, fear about what the doctor might find or other barriers outside the boundaries of this study. This research enhances our understanding of the decision to contact a general practitioner with urological symptoms, and points out that guidelines for PSA testing might be challenged by the high prevalence of urological symptoms.
